# Veterinary costs, productivity and profitability on Norwegian dairy cow and sow farms

**DOI:** 10.1016/j.vas.2026.100613

**Published:** 2026-03-05

**Authors:** Marcel van Asseldonk, Klaus Mittenzwei, Ron Bergevoet, Richard Helliwell

**Affiliations:** a Wageningen University & Research, Wageningen, the Kingdom of the Netherlands; b Institute for Rural and Regional Research, Oslo, Norway

**Keywords:** Animal health economics, Veterinary costs, Antibiotic use, Panel data, Farm accounting

## Abstract

The continuous call for reduced antibiotic use on farms is challenging for farmers without compromising productivity and profitability. In Norway, stringent antibiotic use regulation and disease prevention measures have contributed to achieving the lowest veterinary antibiotic sales in Europe. The aim of this paper is to examine the relationship between veterinary costs (including antibiotic expenditures but also veterinary advice services to support low level of antibiotic use) and farm performance (productivity and profitability) amongst both dairy cow and sow farms in Norway. To examine this relationship, the Farm Accountancy Data Network (FADN) database comprising cross-sectional farm data from 2011 to 2021, was analysed. Average veterinary costs amounted 172 Euro per cow and 84 Euro per sow in 2021. Furthermore, FADN panel data analysis revealed that a higher milk production per cow is associated with higher veterinary costs, namely 1.1 Euro per additional 100 liter per cow. The estimated effect of more piglets sold per sow on veterinary costs amounted approximately 2.48 Euro per extra piglet sold per sow. Dairy and sow farms with higher veterinary costs per animal tend to have lower income per animal. Compared to other European countries livestock productivity is relatively low in Norway while profitability is maintained by means of subsidies easing the need to maximise productivity enabling restrictive antibiotic use.

## 1 Introduction

A key objective for Norwegian agriculture is to maintain low antibiotic use and vigilance on antimicrobial resistance (AMR) through practical measures and developing knowledge to limit the spread of AMR in humans, animals and the environment. Antibiotic use reductions have been a key component of these measures. In 1995 the livestock sector initiated action, in collaboration with public authorities, to improve animal health, prevent disease and reduce antibiotic use by 25 % across all livestock sectors (Animalia, 2017). Since 2000, the Norwegian government has published cross-sectoral plans to address AMR. In 2015, the publication of the National Strategy against Antimicrobial Resistance established a target to reduce antibiotic use in food producing animals by a further 10 % against a 2013 baseline. To support this aim, Animalia, the livestock sector’s umbrella organisation for animal health and welfare, coordinated a joint action plan to support the achievement of this goal (Animalia, 2017). The Norwegian government launched its new 10-year AMR strategy in September 2024. The strategy outlined two objectives especially relevant to agriculture, namely to continue work for stricter prescription guidelines of antimicrobials and increase preventive work to reduce diseases.

As a result of these efforts antibiotic use in Norwegian livestock farming has fallen 53 % between 1995 and 2023 (NORM/NORM-VET, 2024). Furthermore, antibiotic use in Norwegian livestock farming is low in comparison with other Nordic and other European countries as monitored in the European Surveillance of Veterinary Antimicrobial Consumption (ESVAC) database (ESVAC, 2023). In 2022, the veterinary antibiotic use amounted in Norway 7.7 mg of active component per Population Correction Unit (PCU), whereby PCU is a proxy for the biomass of the food-producing animal in a country (ESVAC 2021). Norway has the lowest antibiotic use in the Nordic countries which ranged from 10.6 mg/PCU in Sweden, 14.9 mg/PCU in Finland up to 34.4 mg/PCU in Denmark. The average antibiotic use in 31 European countries was much higher, namely 73.9 mg/PCU (ESVAC, 2023). The numbers for Norway were corrected to cover only terrestrial animals using national statistics (NORM/NORM-VET, 2023) as 75 %of the biomass is farmed fish with very low antibiotic use (0,3 mg/PCU).

While the health benefits of antimicrobial use in animal production are well documented, there is a major lack of information on the farm-level economic impact (Ryan, 2019). The continuous call for reduced current antibiotic use and more stringent animal health measures on farms is challenging for farmers without compromising productivity and profitability (Põldaru & Luik-Lindsaar, 2020; Rojo-Gimeno et al., 2016; Sandeberg et al., 2023). One of the concerns is that (further) decreasing antibiotic use may increase disease incidence, raise animal health costs and, consequently, reduce farm income. Norway offers an interesting case as antibiotic use is substantively lower than most European countries, thus providing an opportunity to investigate the relationship between very low antibiotic use and farm performance.

Veterinary costs comprise both veterinary services and advice as well as medicine costs. These expenses are all direct animal health costs to prevent or cure a disease. The advantage of this Farm Accountancy Data Network (FADN) used aggregated economic indicator is that advice and medicine use can be trade-offs. For example, if antibiotic use is low there is likely a greater need for advice and veterinary services necessary to support low level of antibiotic use.

Eurostat provides an overview of veterinary costs for dairy farms per Member State and as a whole (Eurostat, 2024). Average veterinary costs on dairy farms amounted on average 100 euro^1^ per cow in the EU-27 in 2022 (Eurostat, 2024). In general, the largest part of these costs are related to mastitis treatment amounting approximately to one-third-of the total costs considered (Fourichon et al., 2001). Eurostat does not provide an overview of veterinary costs for other livestock farm types such as pigs and poultry.

Veterinary costs depend on a plethora of factors such as farming system (organic versus conventional production), management practices (e.g., biosecurity and vaccination) and performance level of animals (e.g., milk production per cow and number of piglets per sow). For example, veterinary costs on organic farms are likely reduced because of restricted use of medicines (e.g., antibiotics), although a substitution with other therapies is expected (Agrimatie, 2024; Flaten et al., 2019; KWIN, 2022).

Besides veterinary costs also additional costs are incurred affecting the productivity and profitability of a livestock herd (Hansen et al., 2019). The production effect in diseased animals can result in, for example, premature death, changed value of animals, reduced live weight gain, and reduced yield or quality of products (Dijkhuizen & Morris, 1997). Due to these animal health costs, the economic effect of suboptimal health is substantially higher than the veterinary expenses alone. For example, van Soest et al. (2019) estimated these failure costs of production disorders in organic dairy farms in different European organic dairy herds. They estimated an average loss that varied from 185 euro per dairy cow in Spain to 206 euro in France. Mastitis contributed to the largest part (65 %) of these losses.

In countries starting with a higher use of antimicrobials than Norway both farmers and veterinarians fear that a more restricted use of antimicrobials may have adverse effect livestock production (Coyne et al., 2014). As a guiding prospect it is of interest to study direct veterinary costs, and their relationship with productivity and profitability on Norwegian livestock farms. However, there is a knowledge gap as estimates on the relationship between veterinary costs and overall farm performance Norway are lacking.

The objective of this study is therefore to analyse the relationship between veterinary costs, productivity and profitability on Norwegian farms. This case study is focussing on dairy and sow farms, for which antibiotic use legislation is most relevant in Norway. We exploit data obtained from FADN, which is also the database used by Eurostat to report veterinary costs, productivity and profitability. Insight into this relationship can support policy makers, famers and their advisors in the evaluation of animal health strategies.

## 2 Method

The analysed FADN panel data is an unbalanced farm-level survey collected by the Norwegian Institute of Bioeconomy Research (NIBIO). The selection of FADN farms in Norway is limited to farms where income from agriculture constitutes a significant part of the total income of the farmer's family. Farms are sampled with a minimum threshold of 150,000 Norwegian Krone (NOK)^2^ economic output (NIBIO, 2023, 2025). Participation in the survey is voluntary and there is no limit on the number of years a farm may be included in the survey, however approximately 10 % of the farms surveyed are replaced each year (Sipiläinen et al., 2014). The analysed time period in this study is from 2011 up to and including 2021.

FADN panel data provides general information of farm structure of a typical Norwegian (e.g., dairy and pig) farm, as well as default figures for productivity and profitability. Included in FADN are specialised dairy farms for which >75 % of the farm revenues stems from the dairy operation. Pig farms in Norway are often mixed farms, combining either dairy or cereal production. In the analysis only those pig farms are included from which jointly 75 % of the farm turnover stem from stated two farm activities (i.e., pig and dairy production, or pig and cereal production).

A two-step procedure is followed. We first analyse the veterinary costs per animal as registered in FADN, which include both purchased veterinary services and medicines (i.e., for prevention and cure). Although in FADN purchased veterinary services and medicines are both standard defined variables. challenges arise when decomposing invoices into goods and services delivered. Therefore, we resort to using the more robust aggerated key indicator of veterinary cost per animal (in line with FADN Eurostat reported statistics^3^). Note that in the Norwegian FADN antibiotic dosage is not registered. Nominal veterinary costs (as all other monetary values included in the analysis) are corrected for inflation based on price indices reported by the Norwegian Bank (real values are converted into 2021 levels).

Focussing only on veterinary costs does not include the hidden costs of underperforming animals due to diseases (Hoste & Benus, 2023). A logic step forward is to focus on the relation between veterinary costs and productivity per cow or sow. We examine therefore whether veterinary cost per animal is related to animal performance. The estimated fixed effects model based on the FADN panel data for either dairy or sow farms are defined as follows (Verbeek, 2012):

$$y_{it}=\;\beta_{0}+x_{it}^{{}^{\prime }}\beta_{1}+\;\alpha_{i}+\varepsilon_{it},\;\;\varepsilon_{it}\;∼IID\left(0,\sigma_{\varepsilon }^{2}\right)$$

where $y_{it}$ is the dependent veterinary costs variable of farm i at time t, $\beta_{0}$ is the common intercept term for all subjects over all observations, $x_{it}^{\prime }$ is the vector of independent variables (including among others animal performance) of farm i at time t, $\beta_{1}$ is the vector of coefficients describing the relationship between $x_{it}^{\prime }$ and $y_{it}$ and $\varepsilon_{it}\;$is the observation specific residual term. These residual terms are assumed to follow a normal distribution with a mean of 0 and a constant standard deviation of $\sigma_{\varepsilon }^{2}$. In the case of a fixed effect model, $\alpha_{i}$ is a subject-specific (farm) intercept. The Hausman test is used to determine whether the fixed effect model is more appropriate to use then a random effect model (Verbeek, 2012).

The goodness of fit is measured by means of the within R^2^, the between R^2^ and the overall R^2^. The within R^2^ gives the sh of within-farm variation that is explained by the model. The between R^2^ gives the share of variation between farms that is explained by the model (Wooldridge, 2001).

For the separate dairy and sow regression model the animal performance indicator included milk production per cow or number of piglets sold per sow respectively. Although costs of impaired health could also be substantial in terms of other animal performance indicators (e.g., increased mortality of diseased animals, lower daily gain and feed efficiency for pigs) this was not feasible because of FADN data limitations. Control variables included were the farm’s, farm size, farmer’s age, total labour (including unpaid on-farm labour units as well as paid off-farm labour units of the farmer, spouse and other family members). Year-fixed effects (i.e., year dummies) are included to account for year-specific factors such as market fluctuations, weather impacts, and policy changes.

Subsequently, as a second step, the effect of veterinary costs and productivity variables (i.e., milk production per cow and number of delivered piglets per sow) on total farm income per animal was estimated using the FADN panel. Note that analysing the effect of veterinary costs on farm income is hampered since revenues stem from different activities on Norwegian dairy and sow farms. Based on descriptive statistics 47 % of revenues on dairy farms stem from milk sales, while on mixed sow farms only 28 % of revenues stem from piglet sales. Also gross margin per cow or sow is inherently difficult to assess in FADN since fixed costs, and to a lesser extend variable costs, cannot be allocated to specific activities. This also holds for internal deliveries on the farm (i.e., feed production). In summary, control variables included were farm size, age farmer, labour units (on-farm and off-farm) and year. In summary, this two-step procedure allowed us to view veterinary costs at different levels of farm recordings.

## 3 Results

### 3.1 Descriptive statistics

#### 3.1.1 Performance of dairy farms

In 2021, the FADN panel sample consists of 278 dairy farms and 51 pig farms. Only conventional farms are included in the study, and excluded are approximately 5 % of the Norwegian dairy farms in the presample which are organic, while non of the pig farms apply a organic farming system. Pig farms which are almost equally distributed being either specialised sow, finishing and farrow-to-finish farms. For the remaining of the paper, we focus on farms with sows (i.e., specialised sow and if mentioned specifically also farrow-to-finish farms) since veterinary costs are relatively larger in reproduction than in rearing fattening pigs (Hoste & Benus, 2023). Descriptive statistics of the farms in the 2021 FADN sample including veterinary costs are provided in Table 1. Heterogeneity between years is accounted for in the subsequent panel data analysis.

**Table 1:** Farm characteristics of Norwegian dairy and sow farms in 2021 (mean and standard deviation (Std)).

Dairy farm	Mean	Std	Sow farm	Mean	Std
Dairy cows (number)	35	20	Sows (number)	95	89
Land (hectare)	48	29	Land (hectare)	48	23
Milk production (liter per cow)	6871	1330	Piglets sold (number per sow)	23	6
Unpaid labour (hours)	2771	933	Unpaid labour (hours)	2631	521
Paid labour (hours)	1073	996	Paid labour (hours)	1186	978
Age farmer (years)	49	10	Age farmer (years)	50	10
Farm income (1000 Euro)	93	53	Farm income (1000 Euro)	141	75
Revenues including subsidies (1000 Euro)	260	141	Revenues including subsidies (1000 Euro)	475	218
Subsidies (1000 Euro)	78	32	Subsidies (1000 Euro)	45	26
Fixed costs (1000 Euro)	87	54	Fixed costs (Euro)	220	108
Variable costs (1000 Euro)	80	48	Variable costs (1000 Euro)	114	59
Veterinary costs (1000 Euro)	5.43	3.78	Veterinaryt costs (1000 Euro)	9.35	8.44
Veterinary costs per cow (Euro)	172	96	Veterinary costs per sow (Euro)	84	115

Number of dairy farms in 2021: 278, Number of sow farms in 2021: 15.

The number of cows per farm in the FADN sample amounts on average 35 dairy cows which is (by FADN sampling design) in line with the national average and does not require a weighting procedure. The average cultivated land is 48 hectares. Production per animal amounts on average 6871 L per cow per year. Farm income in 2021 amounts 93.000 Euro per year on dairy farms. Farm income is before depreciation, interest, and paid taxes.

#### 3.1.2 Performance of sow farms

The number sows per farm in the FADN sample amounts 95 on the more specialised sow farms (for all farms with sows the average in the sample amounts 49, which is in line with the national average). The average cultivated land is similar for sow farms as for dairy farms (48 hectares). Production per animal amounts on average 23 piglets sold per sow per year. Farm income in 2021 amounts 141.000 Euro per year on sow farms.

As described before only specialised dairy farms are included in the analyses, while 44 % and 56 % of the pig farms either combine 75 % of their revenues from pig and dairy production or pig and cereal production respectively.

#### 3.1.3 Veterinary costs

On dairy farms, veterinary costs are 5430 Euro per farm and 172 Euro per cow, while these costs are 9350 Euro per sow farm and 84 Euro per sow. For mixed livestock farms (pig and dairy production) the veterinary costs per sow were corrected based on average costs per cow (imputed costs derived from specialised dairy farms) multiplied with the number of dairy cows present. Both on dairy and sow farms veterinary costs per farm and animal are heterogenous when comparing standard deviations with the mean. Note that the median veterinary costs per animal is similar compared to the mean.

Descriptive analysis revealed that the upward trend of real veterinary costs over time increase at a faster pace than productivity. On dairy farms veterinary costs increased in the analysed 11 year time horizon by 66 %, while milk production increased by 11 % (both per cow). Similarly, veterinary costs on sow farms increased by 67 %, while piglet per sow increased by 31 % (both per sow). Time effects in veterinary costs and productivity are analysed in more detail in the panel data regression analysis.

### 3.2 Panel data analysis

The average number of observations per farm in the dairy panel data comprised on average 6.8 years. Because of multicollinearity only one indicator for farm size is allowed in the model (correlation between number of cows and hectares of land is 0.74). The average number of observations per farm in the pig farm panel data comprised on average 4.5 years for sow farms (and almost 6 years for farrow-to-finish farms).

#### 3.2.1 Veterinary costs

##### 3.2.1.1 Dairy farms

Overall, 17 % of the observed variance could be explained by the panel model (Table 2). Significant estimates of the effect of milk production per cow on veterinary costs per cow (*P* < 0.001) amounted 1.1 Euro per 100 L per cow. Controlling for other variables, this study confirms that increasing milk production per cow is associated with an increase in veterinary costs. This is true even in the range of relatively low milk production per cow and low levels of veterinary costs as is the case in Norway.

**Table 2:** Panel data analysis with veterinary costs per cow as dependent variable (Euro)^1^.

	Coefficient	*P*>[t]
Milk production (liter per cow)	0.011	***<0.001
Unpaid labour (hours per cow)	−0.002	0.354
Paid labour (hours per cow)	0.01	0.844
Age farmer (years)	−0.74	***<0.001
Cows (number)	−1.93	***<0.001
2012	4	0.297
2013	12	**0.004
2014	21	***<0.001
2015	26	***<0.001
2016	31	***<0.001
2017	40	***<0.001
2018	32	***<0.001
2019	42	***<0.001
2020	46	***<0.001
2021	43	***<0.001
Intercept	157	***<0.001

1 Subject-specific (farm) intercepts not presented but included in model. R^2^ within 13 %, R^2^ between 17 %, R^2^ overall 17 %. *p < 0.1, **p < 0.05, and ***p < 0.01, Number of observations: 3144, Number of farms: 463.

Allocating more time (i.e., unpaid labour) per cow did not relate to veterinary costs, while older farmers managed their farms in such way that predicted veterinary costs were lower.

Our analysis revealed that farm size matters: a larger herd size (or cultivated land) is associated with lower veterinary cost (1.93 Euro per cow, *P* < 0.001).

Categorical year effects with 2011 as reference year are positive and significant (*P* < 0.001). Note that if specified as trend the predicted veterinary costs corrected for inflation increased 25 Euro per cow per year.

Sow farms

Estimates of the effect of increased piglets sold per sow on veterinary costs per sow amounted approximately 2.48 Euro (*P* = 0.006) per extra piglet sold per sow (Table 2). Similar as on dairy farms, controlling for other variables this study confirms that higher sow productivity is associated with an increase in veterinary costs given the Norwegian context of relative low number of piglets per sow and low levels of veterinairy veterinary costs. Categorical year effects with 2011 as reference year are mostly not significant.

Increasing herd size was associated with lower veterinary cost by 0.5 Euro per sow (*P* = 0.003). Including also farrow-to-finish farms in the analyses had only a marginal effect on estimates. Overall, 9 % of the observed variance could be explained by the alternative models (Table 3). Moreover, the within R^2^ tended to be higher than the overall R^2^ with fixed effects models. This difference was caused by the focus of the fixed effects model on explaining within-subject variation rather than overall variation (Wooldridge, 2001).

**Table 3:** Panel data analysis with veterinary costs per sow as dependent variable (Euro)^1^.

	Coefficient	*P*>[t]
Piglets sold (number per sow)	2.48	***0.006
Unpaid labour (hours per cow)	0.004	0.747
Paid labour (hours per cow)	−0.007	0.545
Age farmer (years)	−1.26	0.159
Sows (number)	−0.50	***0.005
2012	−15.47	0.328
2013	−22.15	0.182
2014	−2.22	0.895
2015	−14.32	0.382
2016	−24.00	0.157
2017	3.83	0.829
2018	−1.40	0.943
2019	−48.64	**0.016
2020	−30.50	0.140
2021	−33.58	0.119
Intercept	137.90	0.078

1 Subject-specific (farm) intercepts not presented but included in model. R^2^ within 24 %, R^2^ between 16 %, R^2^ overall 9 %. *p < 0.1, **p < 0.05, and ***p < 0.01, Number of observations: 227, Number of farms: 51.

Which activity (dairy or cereal production) is combined with pig production only had a limited effect on the estimated animal performance effect. *Re*-estimating by means of a random effect model and including mixed farm types revealed no significant effect of mixed farm type and other estimates were robust.

#### 3.2.2 Farm income

##### 3.2.2.1 Dairy farms

The negative coefficient of veterinary costs per cow was significant (*P* < 0.001) in the income per cow regression model, implying that dairy farms with higher veterinary costs per cow is associated with lower farm incomes per cow (Table 4).

**Table 4:** Panel data analysis with income per cow as dependent variable (Euro)^1^.

	Coefficient	*P*>[t]
Veterinary costs (Euro per cow)	−1.71	***<0.001
Milk production (liter per cow)	0.26	***<0.001
Unpaid labour (hours per cow)	5.35	0.533
Paid labour (hours per cow)	−0.97	***<0.001
Age farmer (years)	−4.69	***<0.001
Cows (number)	−18	**0.013
2012	365	***<0.001
2013	295	***<0.001
2014	328	***<0.001
2015	547	***<0.001
2016	550	***<0.001
2017	514	***<0.001
2018	376	***<0.001
2019	300	***<0.001
2020	314	***<0.001
2021	623	***<0.001
Intercept	1080	***<0.001

1 Subject-specific (farm) intercepts not presented but included in model. R^2^ within 26 %, R^2^ between 28 %, R^2^ overall 27 %. *p < 0.1, **p < 0.05, and ***p < 0.01, Number of observations: 3400, Number of farms: 496.

Moreover, increased livestock productivity (i.e., milk production per cow) was associated with a higher farm income (*P* < 0.001). Most other included independent variables were significant. For example, categorical year effects with 2011 as reference year are positive and significant (*P* < 0.001). Note that if specified as trend the predicted income per cow (corrected for inflation) increased 25 Euro per cow per year.

Sow farms

The estimated coefficient of veterinary costs per animal was significant on sow farms (Table 5). Increased livestock productivity (i.e., piglets sold per sow) was associated with a higher income per sow. In this panel-data regression model the year variable was important: higher farm incomes were obtained in years with higher piglet prices (and vice versa). The volatility in market prices in the pig sector stems from the so-called pig cycle, i.e. cyclical fluctuations in the market due to delayed supply responses to price changes (Harlow, 1960).

**Table 5:** Panel data analysis with income per sow as dependent variable (Euro)^1^.

	Coefficient	*P*>[t]
Veterinary costs (Euro per sow)	−2.23	***0.002
Piglets sold (number per sow)	52	***<0.001
Unpaid labour (hours per sow)	24	***<0.001
Paid labour (hours per sow)	−3.84	0.283
Age farmer (years)	5.27	0.487
Sows (number)	−2.80	*0.093
2012	44	***<0.001
2013	−328	***<0.001
2014	−133	0.752
2015	275	**0.026
2016	176	0.370
2017	84	*0.057
2018	−416	0.243
2019	−35	0.591
2020	314	**0.016
2021	574	0.849
Intercept	−526	0.356

1 Subject-specific (farm) intercepts not presented but included in model. R^2^ within 52 %, R^2^ between 55 %, R^2^ overall 46 %. *p < 0.1, **p < 0.05, and ***p < 0.01, Number of observations: 212, Number of farms: 47.

## 4 Discussion

In this paper the relationship between veterinary costs and farm performance (productivity and profitability) amongst both dairy cow and sow farms in Norway was examined by using FADN panel data. Higher veterinary costs were not offset by lower disease costs, which could be explained by the fact that farmers who are less in control of disease management are also less in control of other farm operational activities. Because of FADN data limitations the underlying dynamics cannot be assessed by means of the current available variables. For example, measures taken on each farm to improve animal health are not collected and requires additional farm interviews (Van Asseldonk et al., 2020).

The analysis is also context specific. Compared to other European countries livestock productivity is low in Norway while profitability is maintained by means of subsidies easing the need to maximise productivity enabling restrictive antibiotic use. Norwegion dairy farms are typical small scale in terms of the number of dairy cows compared to most the top four milk-producing countries in the EU (Germany, France, The Netherlands and Italy). Only in Poland the average number of cows per farm were less (21 dairy cows in 2021), while in Italy, France, Germany and The Netherlands the average herdsize was 70, 74, 80, and 106 dairy cows respectively (Parzonko, 2024). With respect to dairy performance in terms of milk production per cow it is at the lower tail of the general European production level, while substantial heterogeneity exists between countries. The highest and lowest milk production was obtained in Sweden and Bulgaria with on average 10,379 and 3555 L per cow respectively (Eurostat, 2024). Also sow farms are typical small scale in terms of the average number of sows present compared to key exporting countries in Europe (i.e., Denmark and the Netherlands for which average number of sows exceeds 800). With respect 23 piglets sold per sow it is substantial lower than high performing countries like Denmark and The Netherlands in which piglets sold per sow exceed 30–35 per year (Hoste & Benus, 2023).

Is it difficult to benchmark Norwegian veterinary costs and compare the estimated relationship between veterinary costs, productivity and profitability with other European countries. Eurostat only provides an overview of veterinary costs for dairy farms and not for sow farms, and moreover only averages hampering a between farm within country analysis (Eurostat, 2024). Exploiting other data sources for international benchmarking purposes is quite a challenge because of different definitions (i.e., semantics) on which costing components are exactly captured (Hoste & Benus, 2023). Moreover, in the Norwegian FADN, antibiotic use and related antibiotic treatment costs are not registered and they are not required in the EU FADN data collection procedure. In fact up to now only a few FADN Liasson agencies enrich FADN data with antibiotic use data as is the case in the Netherlands (Van Asseldonk et al., 2020) and Denmark. To make an international comparison beyond current collected FADN veterinary costs a more extensive and harmonised approach is therefore necessary (including antibiotic use and antibiotic treatment costs).

FADN is currently converting into the Farm Sustainability Data Network (FSDN). FSDN is regarded as a critical step in a more comprehensive understanding of the EU progress towards sustainability (European Commission, 2024). In the Farm to Fork strategy the target is to reduce overall EU veterinary antibiotic sales by 50 % by 2030. Moreover, improving animal welfare standards is one of key sustainable objectives as it may contribute to improving livestock health, reducing the need for antibiotic treatments and thus decreasing the risk of AMR (Diana et al., 2020). The One Health action plan against AMR aims to preserve the possibility of effective treatment of infections in humans and animals (European Commission, 2020). Antimicrobial resistance is addressed through regulations on veterinary medicinal products and medicated feed, promoting responsible antimicrobial use, as well as through specific technical guidelines.

The feasibility to include antibiotic use per species of animal and product in FSDN is under investigation (European Commission, 2024). For most EU Member States that do not currently collect antibiotic use data, initiating such data collection is a substantial burden, especially considering the required level of detail (i.e., breaking down invoices from veterinarians to identify the required information, such as recognising and categorising antibiotics products). However, FADN Liaison agencies in Denmark and the Netherlands have implemented a data collection process requiring minimal effort by extracting antibiotic use from other digital databases (through official online logbooks from pharmacies and veterinary practitioners). This additional data will enable to estimate the impact of policy reforms on antibiotic use, veterinary costs and farm income.

For a more meaningful in-depth impact analysis of antibiotic use reduction strategies on productivity and profitability by means of the current FADN or prospective FSDN variables will remain hampered. Many management practices to reduce antibiotic use, such as improved biosecurity (Kruse et al., 2018, 2019) and the level of technology implementation (Van Asseldonk et al., 2020) are difficult to operationalise and not included in EU FADN (FSDN) recordings. Also behavioural factors, such as intentions, attitudes, beliefs and perceptions, that explain the behaviour response to keep or get the use of antibiotics under the target value requires a complementing survey among farmers in the panel (Van Asseldonk et al., 2020).

Compared to other European countries livestock productivity is low in Norway while profitability is maintained by means of subsidies easing the need to maximise productivity and enabling restrictive antibiotic use. Norway has retained a high level of political control and fiscal support for agriculture in contrast to the significant liberalisation of agricultural policy and declining financial support that occurred across EU Member States. As a member of the European Economic Area Norway retains sovereignty over trade policy and agricultural policy and is not a part of the Common Agricultural Policy (OECD, 2021). Sovereignty has been central to past and present success in restrictive antibiotic use and AMR governance (Finstad & Fuglestad, 2024). Norway is likely to retain national sovereignty over its agricultural policy which would remain significantly interventionist and protectionist. However, the financial framework conditions are a key uncertainty and Norwegian agricultural policy continues to drive a trend of farm consolidation and up-scaling alongside per animal livestock productivity increases.

## 5 Conclusion

There is worldwide emphasis on reducing antibiotic usage in livestock production (EU, 2024; FAO, 2021; WOAH, 2022). Norway antibiotic use legislation is one of the most stringent ones in the world. The result has been that Norway has become an international forerunner, and has successfully maintained a high health, high welfare agricultural regime with low antibiotic use and low AMR prevalence (Finstad & Fuglestad, 2024). However, farm productivity in Norway is low compared to countries with similar economic development. The continuous call for reduced antibiotic use on farms is challenging for farmers without compromising productivity and profitability. As a guiding prospect the objective of this study was to analyse the relationship between veterinary costs, productivity and profitability on Norwegian farms. In this study we quantify that increasing livestock productivity will also increase veterinary costs. Compared to other European countries animal productivity is low in Norway while profitability is maintained by means of subsidies easing the need to optimise productivity (enabling low veterinary costs and restrictive antibiotic use). However, despite subsidies Norwegian agricultural policies have continued to drive a trend of sector consolidation and livestock productivity increases. If this trend continues, as seems likely, it will put even more emphasis on increasing productivity and thus, may inter alia, affect veterinary costs in the future.

## Funding

The project LIMBO: Evaluating emerging AMR threats and future capacity for action in Norwegian livestock agriculture was financed by the Research Council of Norway.

## Data availability statement

The FADN data that support the findings of this study are confidential and were obtained from the national liaison agencies under strict, regulated access conditions. Researchers can request access via the official FADN website (DG-Agri, European Commission) or national liaison agencies.

## Ethical

FADN processing activities in this study are conducted in strict compliance with the EU General Data Protection Regulation (GDPR).

## CRediT authorship contribution statement

**Marcel van Asseldonk:** Writing – original draft. **Klaus Mittenzwei:** Writing – review & editing. **Ron Bergevoet:** Writing – review & editing. **Richard Helliwell:** Writing – review & editing, Project administration.

## Declaration of competing interest

The authors declare that they have no known competing financial interests or personal relationships that could have appeared to influence the work reported in this paper.

## Footnotes

1 Exchange rate 1.0801 euro/USD in 2024:, source European Central Bank.

2 Exchange rate 11.6276 NOK/Euro in 2024, source Norges Bank.

3 https://agridata.ec.europa.eu/extensions/FarmEconomyFocus/FADNDatabase.html
